# Intensity-Modulated Radiation Therapy for Esthesioneuroblastoma: 10-Year Experience of a Single Institute

**DOI:** 10.3389/fonc.2020.01158

**Published:** 2020-07-17

**Authors:** Cihang Bao, Weixu Hu, Jiyi Hu, Yuanli Dong, Jiade J. Lu, Lin Kong

**Affiliations:** ^1^Department of Radiation Oncology, Fudan University Shanghai Cancer Center, Shanghai, China; ^2^Department of Radiation Oncology, Shanghai Proton and Heavy Ion Center, Shanghai, China; ^3^Shanghai Engineering Research Center of Proton and Heavy Ion Radiation Therapy, Shanghai, China

**Keywords:** intensity-modulated radiotherapy, esthesioneuroblastoma, elective nodal irradiation, toxicities, outcomes

## Abstract

**Objectives:** To evaluate efficacy and safety of intensity-modulated radiotherapy (IMRT) in the management of esthesioneuroblastoma (ENB).

**Methods and Materials:** A retrospectively analysis of 52 ENB patients treated with IMRT between 8/2008 and 8/2018 was performed. Thirteen of the 44 patients (29.5%) with newly diagnosed and 2 of the 8 patients with recurrent disease presented regional lymph node metastasis. The median dose of IMRT was 66 (range 52.5–75) Gy for all patients. Elective nodal irradiation (ENI) was provided to all excluding 6 patients in this cohort.

**Results:** With a median follow-up time of 32.5 (6~121) months, the 3-year overall survival (OS), progression-free survival (PFS), local progression-free survival (LPFS), regional progression-free survival (RPFS), and distant metastasis-free survival (DMFS) rates for the entire cohort were 89.7, 69.5, 89.7, 95.1, and 85.4%, respectively. Multivariate analysis revealed that N-classification (N– vs. N+) at presentation was the only significant prognosticators for PFS. No significant prognosticator was identified for other survival outcome. No severe (i.e., grade 3 or 4) IMRT-induced acute toxicity was observed. Severe late toxicities were infrequent (11.5%), which included dysosmia (3.8%), hearing loss (3.8%), radiation brain injury (1.9%), and temporal lobe necrosis (1.9%). Moreover, late ocular toxicity secondary to IMRT was not observed.

**Conclusion:** IMRT produced acceptable 3-year outcomes in terms of OS (89.7%), LPFS (89.7%), and RPFS (95.1%) rates without substantial late adverse effects. Further investigations for a more effective systemic strategy for distant disease control as well as a precision radiation technique for further improvement in local control are needed.

## Introduction

Esthesioneuroblastoma (ENB), also known as olfactory neuroblastoma, is an uncommon malignancy of neuroectodermal origin and constitutes 3% of all intranasal neoplasms (1). Due to its rarity and heterogeneous biological behavior, no uniformly accepted standard treatment has been established. Although surgery is generally accepted as the initial treatment of choice, complete resection with sufficient margins is often challenging due to the anatomical location of ENB. Radiation therapy, either with definitive (radiotherapy exclusively or radiochemotherapy) or adjuvant intention, is a vital component of the multidiscipline management of the disease. Results of retrospective series has demonstrated that adjuvant radiotherapy after surgery was effective in improving local control (2–7). Furthermore, high-dose radiotherapy offers the only potential for cure for unresectable or inoperable ENB, and may provide similar outcome as compared to surgery for early stage diseases (6). Nevertheless, the dose of radiation is usually limited by critical organs at risk (OARs) usually within the radiation field, especially for locally advanced ENB with intracranial extension.

Intensity-modulated radiation therapy (IMRT) offers the potential to reduce dose to OARs while maintaining doses at therapeutic levels to the target volumes via optimized non-uniform beam intensities. The benefit of IMRT is particularly profound in the management of sinonasal tumors which are usually irregular in contours and located in the immediate vicinity of vital neurological and vascular structures (8–10). However, the use of IMRT for the management of ENB has yet to be studied further. The aim of this study is to document the outcome of a relatively large group of patients with ENB treated in a uniform fashion with IMRT.

## Methods and Materials

This retrospective study was approved by the institutional review board (IRB) of the Fudan University, Shanghai Cancer Center (FUSCC), and all patients provided written informed consent for medical research prior to initial treatment.

### Patients' Criteria

Between 8/2008 and 8/2018, 57 consecutive patients with histologically confirmed ENB were treated at the FUSCC. One patient lost in follow-up immediately after the completion of IMRT and 4 patients refused to receive IMRT due to financial reasons were excluded from this analysis. For the remaining 52 patients (Table 1), their extent of disease was determined by review of CT or MRI of the head and neck as well as surgical reports. Tumor stage was evaluated and confirmed using the modified Kadish staging system (11). Hyams grade were available for only 12 patients. The gross tumor volume (GTV) was defined based on the diagnostic CT and/or MRI. Surgical tumor bed of patients underwent resection were also included in GTV. The clinical target volume (CTV) was defined as the GTV plus a margin for subclinical diseases as well as the draining lymphatics in the neck. Elective nodal irradiation (ENI), which covered the primary tumor as well as the draining lymphatics in the neck, was provided to all patients excluding 6 patients in this cohort. The use of induction and/or adjuvant chemotherapy was at the discretion of their referring medical oncologists. All patients treated with chemotherapy received two or more cycles.

**Table 1:** Characteristics of 52 patients with Esthesioneuroblastoma.

	**Primary IMRT** **(44 patients)**		**Salvage IMRT** **(8 patients)**	
	***n***	**%**	***n***	**%**
**Gender**				
Male	35	79.5	6	75
Female	9	20.5	2	25
**Age (years)**				
Median(range)	44 (18–67)		64 (55–74)	
**Modified Kadish stage**				
A	4	9.1	0	0
B	12	27.3	5	62.5
C	15	34.1	1	12.5
D^*^	13	29.5	2	25
**T-classification**				
1	10	22.7	3	37.5
2	15	34.1	3	37.5
3	8	18.2	2	25
4	11	25	0	0
**N-classification**				
0	31	70.5	6	75
1	13	29.5	2	25
**Surgery**				
R0/R1	10	22.7	2	25
R2	20	45.5	3	37.5
Biopsy	14	31.8	3	37.5
**Chemotherapy**				
No	20	45.5	4	50
Yes	24	54.5	4	50
**Total dose of IMRT, Gy**				
Median (range)	66 (52.5–75)		66 (56–66)	
**Fractionation of IMRT, Gy**				
Median (range)	2 (1.8–2.2)		2 (2–2.2)	
**ENI**				
No	2	4.5	4	50
Yes	42	95.5	4	50

* *All had regional lymphadenopathy without distant metastasis. IMRT, intensity-modulated radiation therapy*.

### Statistics

Time to local, regional, and distant failure as well as death were estimated from the date of diagnosis of disease or recurrence until documented event. Univariate analyses for survivals were performed using Kaplan-Meier method (with the log-rank test) and the univariate Cox proportional hazards model. The prognostic factors were determined by the multivariate Cox proportional hazards model. Statistical calculation was performed with SPSS (version 19.0) and R software (version 3.5.3) was used to draw survival curves. *P* values < 0.05 were considered statistically significant.

## Results

### Baseline Characteristics

The characteristics of the patients and their treatment strategy are detailed in Table 1. Fifteen patients presented with neck adenopathy including 2 failed a previous course of radiation. The characteristics of the regional (neck node) metastases in the 13 patients with newly diagnosed ENB is detailed in Figure S1. The neck nodal stations were classified according to the DAHANCA, EORTC, GORTEC, NCIC, and RTOG consensus (12). Bilateral neck adenopathy was seen in 6 (46.2%) among these 13 patients. One patient presented with skip metastasis, and the remaining presented in a contiguous pattern. Level IV and V nodes were implicated only in case with disease widely metastatic to the upper and middle neck nodes.

Thirty-five patients underwent surgery including 10 with endoscopic resection. Twelve patients achieved R0 or R1 resection, and the remaining 23 had partial resection (Table 1). Elective neck dissection was not performed unless for patients with known neck adenopathy. The remaining 17 patients received biopsy only.

Forty-four patients received primary IMRT (newly diagnosed patients received first course of IMRT), and 1 patient with local recurrence after surgery alone received high-dose salvage IMRT. In addition, 4 patients failed previous radiotherapy (non-IMRT) for ENB and 3 patients with radiation-induced ENB as second primary tumor after treatment for nasopharyngeal cancer (*n* = 2) or nasal NK/T cell lymphoma (*n* = 1) received a second course of radiation using IMRT. The latent period between the 2 courses of radiotherapy for all 7 patients were > 3 years.

For 44 patients who received primary IMRT, the total dose to the CTV of covers the GTV ranged from 52.5 to 75 (median = 66) Gy in conventional fractionations (1.8~2.2 Gy per daily fraction). Two patients with stage C and D disease, respectively, discontinued IMRT due to adverse effects at 52.5 Gy (2.1Gy/daily fraction) and 56 Gy (2 Gy/daily fraction). The doses of ENI were 60 Gy for 37 patients, and were 50~54 Gy for 5 additional patients. Two patients did not receive ENI.

For the 8 patients who received salvage IMRT (1 with local recurrence after surgery, 4 failed previous course of radiotherapy, and 3 with radiation-induced second primary ENB), the total dose to the CTV ranged from 56 to 66 (median = 66) Gy at 2.0~2.2 Gy/daily fraction. One patient received 56 Gy in 28 fractions after R0/R1 resection. Two patients received 60 Gy, and the remaining 5 received 66 Gy. Four patients received ENI with the doses at 50, 60, 60, and 66 Gy. The remaining 4 patients received IMRT to the primary lesions only.

Induction chemotherapy were provided to 13 patients, and the most commonly used regimen was etoposide and cisplatin (EP). Ten patients received platinum-based chemotherapy in concurrent with IMRT. And 6 patients received adjuvant chemotherapy (platinum + etoposide, gemcitabine, or etoposice/cyclophosphamide) after the completion of IMRT.

### Disease Control and Survival

The median follow-up time for the entire cohort of 52 patients was 32.5 (6~121) months. Three and two patients received primary or salvage IMRT, respectively, had deceased. The 3-year overall survival (OS), progression-free survival (PFS), local progression-free survival (LPFS), regional progression-free survival (RPFS), and distant metastasis-free survival (DMFS) rates for the entire cohort were 89.7, 69.5, 89.7, 95.1, and 85.4%, respectively.

### Patterns of Failure

Among the 44 patients treated with primary IMRT, only 2 patients with modified Kadish Stage C disease (T3N0M0) experienced local failure. In addition, 2 patients with stage D (N1) experienced regional failure. Furthermore, 4 patients developed distant metastases in bone, lung, and/or distant nodal region at 7, 8, 13, and 23 months after the completion of IMRT. Among the 8 patients received salvage IMRT, 4 patients developed local (2 patients) and distant (2 patients) failure, respectively. The patterns and details of failure for all patients are demonstrated in Table 2.

**Table 2:** Details of the 12 patients who experienced local and/or distant failures and their treatment.

**Gender/age (y)**	**KS**	**Neck**	**Nature of RT**	**Treatment received**	**RT Dose (Gy)**	**Failure (mo)**	**Salvage at failure**	**Final status [last follow-up time, mo]**
M/33	C	N0	Primary	S+CT+RT	66	LR (12)	CT	AWD (21)
M/53	C	N0	Primary	S+CT+RT	66	LR (20)	S+CT	AWD (25)
M/41	D	N1	Primary	S+CT+CCRT	66	RR (24)	CT	AWD (29)
M/40	D	N1	Primary	RT	70	RR (5)	S	NED (72)
F/35	B	N0	Primary	S+RT	66	DM (13)	CT	DOD (30)
M/29	C	N0	Primary	S+RT	59.4	DM (7)	RT+CT+RT	DOD (23)
F/46	D	N1	Primary	S+RT	66	DM (23)	RT+CT	AWD (38)
M/64	D	N1	Primary	CT+CCRT+CT	66	DM (7)	CT	AWD (8)
M/64	B	N0	Salvage	S+CT+RT	60	LR (16)	None	AWD (24)
M/64	D	N1	Salvage	CT+RT	66	LR (25)	Unknown	AWD (25)
M/71	B	N0	Salvage	S+RT	60	DM (7)	None	DOD (8)
F/55	D	N1	Salvage	CT+CCRT	66	DM (30)	S	AWD (81)

*The last 4 patients received salvage IMRT. KS, modified Kadish stage; RT, radiotherapy; S, surgery; DM, distant metastasis; LR, local recurrence; RR, regional recurrence; NED, no evidence of disease; DOD, death from disease; CT, systemic-dose chemotherapy; CCRT, concurrent chemoradiation. AWD, alive with disease*.

### Prognostic Factors

All significant prognosticators, previously reported in the literatures for ENB after radiotherapy for local and regional disease control were assessed in both univariate and multivariate analyses for this cohort of patients (Tables 3, 4 and Tables S1–S5). These potential prognosticators included age, gender, recurrent vs. initial diagnosis, salvage vs. primary IMRT, modified Kadish stage, Dulguerov T- and N-classifications, use of chemotherapy or surgery, dose of IMRT to GTV, fractionation, biological equivalent dose (BED), and use of ENI.

**Table 3:** Univariate analyses for survival outcomes by Kaplan-Meier method (log-rank).

**Variables**	**OS**	**PFS**	**LPFS**	**RPFS**	**DMFS**
Gender (female vs. male)	0.771	0.784	0.224	0.403	0.148
Age (≤ vs. > 50)	0.397	0.343	0.205	0.211	0.796
Recurrent ENB (no vs. yes)	**0.035**	**0.012**	0.213	0.650	**0.030**
Salvage RT 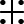 (no vs. yes)	0.163	**0.038**	**0.045**	0.548	0.234
Modified Kadish stage (A/B/C vs. D)	0.748	**0.018**	0.992	**0.018**	0.149
T-category (T1/2 vs. T3/4)	0.350	0.629	0.171	0.805	0.243
N-category (N– vs. N+)	0.748	**0.018**	0.992	**0.018**	0.149
Surgery (No vs. Yes)	0.765	0.351	0.809	0.573	0.941
Chemotherapy^*^(No vs. Yes)	0.227	0.587	**0.032**	0.974	0.403
GTV dose (< vs. ≥66 Gy)	0.551	0.643	0.947	0.431	0.662
Fractionation (< vs. ≥2.1 Gy)	0.990	0.663	0.388	0.490	0.944
BED (≤ vs. >79.2 Gy)	0.880	0.941	0.313	0.332	0.759
ENI (No vs. Yes)	0.143	0.118	**0.019**	0.604	0.727

* Chemotherapy before RT and/or concurrent chemotherapy and/or Chemotherapy after RT.

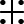
Salvage RT including 1 local recurrence after surgery and 7 re-irradiation patients.

Bold values indicates P < or ~0.05, which was considered statistically significant.

**Table 4:** Multivariate analyses of PFS (Cox proportional hazards model).

**Variables**	**Multivariate analyses**	
	**HR (95% CI)**	***P*-value**
Gender (female vs. male)	1.590 (0.316–7.993)	0.574
Age (continuous variable)	1.007 (0.958–1.058)	0.797
Salvage RT 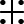 (no vs. yes)	2.895 (0.279–30.005)	0.373
T-category (T1/2 vs. T3/4)	0.312 (0.071–1.375)	0.124
N-category (N– vs. N+)^‡^	4.774 (1.388–16.423)	**0.013**
Surgery (No vs. Yes)	0.900 (0.212–3.824)	0.886
Chemotherapy^*^ (No vs. Yes)	1.277 (0.326–4.998)	0.726
GTV dose (continuous variable)	1.124 (0.902–1.400)	0.299
Fractionation (continuous variable)	0.000 (0.000–3.206)	**0.077**
ENI (No vs. Yes)	1.065 (0.092–12.276)	0.960

* Chemotherapy before IMRT and/or concurrent chemo-IMRT, and/or Chemotherapy after IMRT.

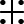
Salvage RT including 1 local recurrence after surgery and 7 re-irradiation patients.

‡ Constant or Linearly Dependent covariates Modified Kadish stage (A/B/C vs. D) = N stage (N− vs. N+).

Bold values indicates P < or ~0.05, which was considered statistically significant.

**Figure 1:**
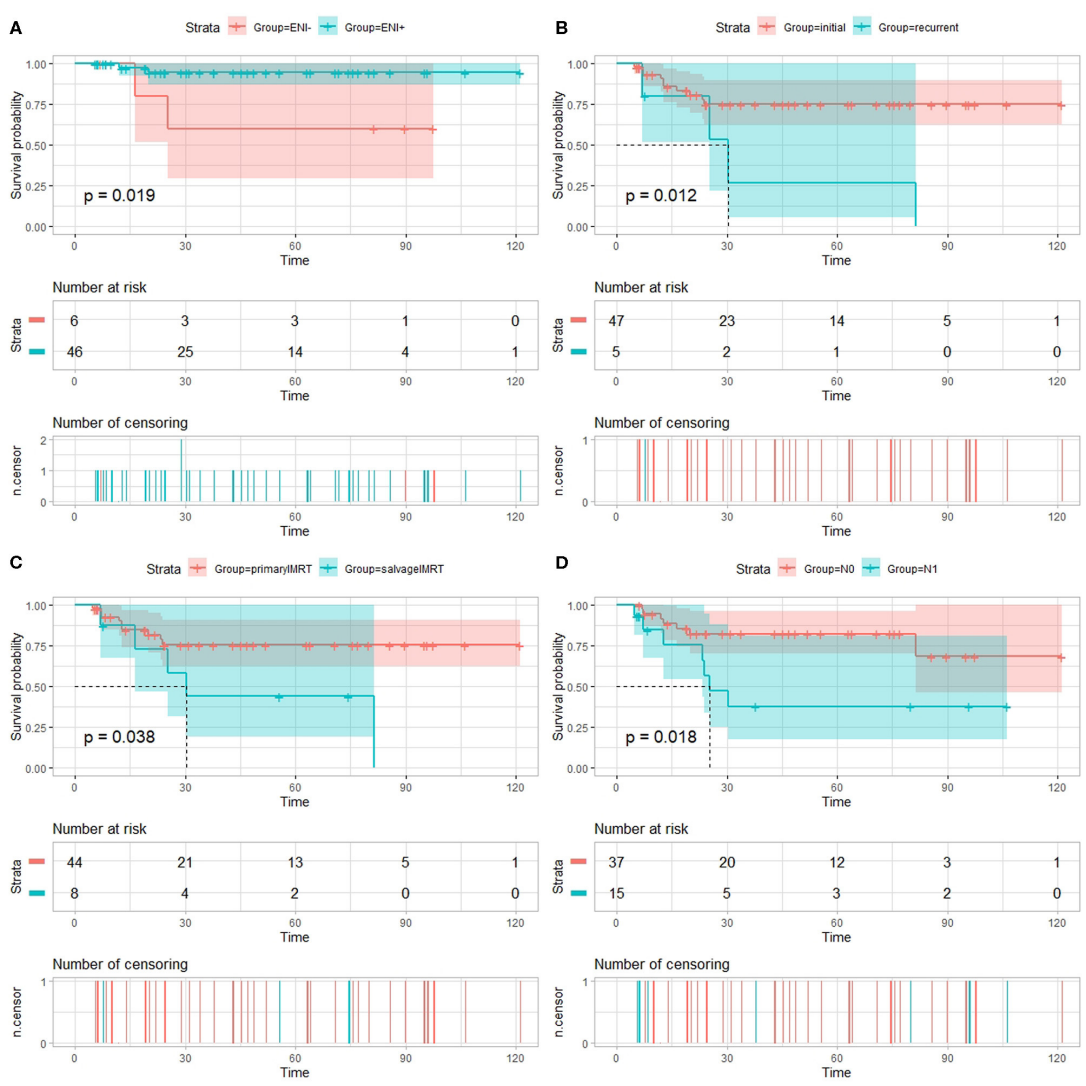
Univariate analysis using the log-rank test. **(A)** Local progression-free survival (LPFS) of patients with esthesioneuroblastoma treated with intensity-modulated radiotherapy (IMRT) stratified by use of elective nodal irradiation (ENI). Progression-free survival (PFS) of patients with esthesioneuroblastoma treated with IMRT stratified by recurrence **(B)**, salvage IMRT **(C)**, and N-classification **(D)**.

On univariate analysis using the log-rank test (Table 3), the use of ENI significantly improves LPFS (*p* = 0.019, Figure 1A); recurrence was a significant prognosticator for both DMFS (*p* = 0.030), PFS (*p* = 0.012, Figure 1B), and OS (*p* = 0.035); salvage IMRT is a significant predictor for PFS (*p* = 0.038, Figure 1C) and LPFS (*p* = 0.045); Patients receiving chemotherapy experienced worse LPFS (*p* = 0.032). Univariate analyses using the cox regression analysis (Tables S1–S5) revealed that recurrence was a significant prognosticator for PFS (HR, 3.986; 95% CI: 1.246–12.754, *p* = 0.020); salvage IMRT is a significant predictor for PFS (HR, 3.009; 95% CI: 1.007–8.991, *p* = 0.049); the use of ENI significantly improves LPFS (HR, 0.136; 95% CI: 0.019–0.964, *p* = 0.046); recurrence showed a trend to predict DMFS (HR, 5.373; 95% CI: 0.981–29.426, *p* = 0.053), and OS (HR, 5.886; 95% CI: 0.931–37.231, *p* = 0.060); salvage IMRT showed a trend to predict LPFS (HR, 5.840; 95% CI: 0.821–41.522, *p* = 0.078). However, no significant association between chemotherapy and LPFS was observed on cox regression analysis (*p* = 0.293, Table S3).

Patients diagnosed with N1 stage have worse RPFS than those with N0 stage (81.7 vs. 100%, log-rank: *p* = 0.018), while no significant association between N-classification and RPFS was observed on cox regression analysis (*p* = 0.464, Table S4). Moreover, the 3-year PFS rates for the 37 patients diagnosed with N0 (median PFS not reached) vs. the 15 patients diagnosed with N1 (median PFS 25.4 months) were 82.2 vs. 37.7%, respectively (log-rank: *p* = 0.018, Figure 1D; Cox: HR, 3.295, 95% CI: 1.154–9.408, *p* = 0.026, Table S2).

No significant prognosticator was found in multivariate analyses for survival outcomes except for PFS (Table 4). Modified Kadish stage (A/B/C vs. D) and N-classification (N– vs. N+) were constant or linearly dependent covariates. And BED was calculated from total dose (to GTV) and fractionation. Therefore, potential prognostic factors, including age, gender, salvage vs. primary IMRT, Dulguerov T- and N-classifications, use of chemotherapy or surgery, dose of IMRT to GTV, fractionation and use of ENI were included in the multivariate analysis. The only independent factors predicting PFS were N-classification (N– vs. N+) (HR, 4.774, 95% CI: 1.388–16.423, *p* = 0.013). Fractionation of IMRT showed a trend to predict PFS (*p* = 0.077).

### Radiation-Induced Adverse Effects

Acute Radiation Morbidity Scoring Criteria of the Radiation Therapy Oncology Group (RTOG) was used for IMRT-induced acute toxicities that observed within 3 months after the initiation of IMRT. Grade 1-2 acute toxicities were observed in most patients, which included mucositis, dermatitis, neutropenia, anemia, or thrombocytopenia. The most commonly observed acute toxicity was mucositis. Four, 12, 11, and 1 patient respectively experienced Grade 1, 2, 3, and 4 mucositis but recovered after supportive care. No other patient experienced Grade 3 or 4 acute toxicity otherwise.

Late toxicities included those occurred 3 months after the initiation of IMRT and were assessed using the Late Radiation Morbidity Scoring Criteria of the RTOG (13) and were detailed in Table 5. Fifteen patients reported IMRT-related late toxicities at their last follow-up. 11.5% of 52 ENB patients presented severe late toxicities were treated by IMRT with (7.7%) and without (3.8%) chemotherapy (*p* = 0.815, Table S6), respectively.

**Table 5:** Type, severity, and frequency of late toxicities.

**Toxicity**	**IMRT with** **chemotherapy**		**IMRT without chemotherapy**	
	**Grade 1 or 2**	**Grade ≥ 3**	**Grade 1 or 2**	**Grade ≥ 3**
	**No. of patients (%)**	**No. of patients (%)**	**No. of patients (%)**	**No. of patients (%)**
Nasopharyngeal Mucositis	0 (0%)	0 (0%)	0 (0%)	0 (0%)
Temporal lobe necrosis	0 (0%)	1 (1.9%)^*^	0 (0%)	0 (0%)
Radiation brain injury	5 (9.6%)	1 (1.9%)	0 (0%)	0 (0%)
Xerostomia	1 (1.9%)	0 (0%)	4 (7.6%)	0 (0%)
CNN				
Hearing Loss	0 (0%)	1 (1.9%)^*^	0 (0%)	1 (1.9%)^*^
Visual acuity	2 (3.8%)	0 (0%)	0 (0%)	0 (0%)
Dysosmia	0 (0%)	1 (1.9%)	0 (0%)	1 (1.9%)

* *Patients received re-irradiation. CNN, Cranial nerve neuropathy*.

## Discussion

In this retrospective analysis, we studied 52 ENB patients most with locally advanced and unresected disease. All patients received IMRT (median dose = 66 Gy) with (88.5%) or without (11.5%) ENI. The 3-year OS, LPFS, RPFS, and PFS rates were 89.7. 89.7, 95.1, and 69.5%, respectively. The OS and PFS from our analyses were comparable or slightly superior as compared to those previously reported (7, 14, 15). However, severe late adverse effects after IMRT with or without chemotherapy were infrequent, which included dysosmia (3.8%), hearing loss (3.8%), radiation brain injury (1.9%), and temporal lobe necrosis (1.9%). Not surprisingly, patients who presented neck node metastasis had worse outcome in terms of PFS on multivariate analyses.

ENB is a relatively rare malignancy in the head and neck, and radiotherapy is an important modality for its management. Due to its high local failure rate, adjuvant radiation after surgery has been shown to improve local control of the disease and potentially survival, especially for locally advanced diseases (16–18). For patients with inoperable or unresectable diseases, high-dose radiation therapy is the only curative treatment modality. However, despite of its efficacy for early stage disease, local control for locally advanced ENB was suboptimal. In a retrospective study of 55 patients, Benfari et al. reported local control rates of 58 and 19%, respectively, for Kadish B and C patients (19). Such dismal outcome was due to, at least in part, the lower dose (median dose = 55 Gy) used. More recently published clinical results have indicated that the use of conformal techniques like IMRT or proton beam therapy at higher doses may improve outcomes for local control and minimizing radiation-induced adverse effects to the nearby OARs (20–22).

Although a dose-response has not been confirmed for ENB, higher radiation dose, in theory, may improve local control thereby overall outcome. Owing to its initial inconspicuous location and unspecific symptoms (primarily nasal obstruction with or without recurrent epistaxis) (14, 23–27), ENB is often locally advanced (frequently extended into the orbits, sinuses, and anterior cranial fossa) at diagnosis. As such, the dose of conventional radiotherapy is often substantially limited by the OARs. The use of precision radiation therapy such as IMRT and proton therapy have the physical advantage in improving therapeutic ratio. In a retrospective of 116 patients reported by Yin et al., the use of 2D vs. 3D or IMRT produced similar outcome in term of LPFS, DMFS, and OS (28). However, radiation-induced adverse effects cannot be ignored and usually arrange between 30 and 40% (16). In fact, sinonasal radiotherapy is challenging due to the close anatomical association between the tumor bed and OARs including eye, optic pathway, brain and brainstem. The incidence of unilateral and bilateral grade 3–4 radiation-induced retinopathy and optic neuropathy, for instance, reported to be as high as 30 and 10% respectively (29) after conventional radiation. In a study used 3D-CRT, 9% of patients developed RT-related severe late toxicity (23), suggesting more precise radiation technique may improve the toxicity profile after radiotherapy. The efficacy of IMRT for tumor in nasal cavity and paranasal sinuses including 7 ENB patients reported by Daly et al. (30) suggested that IMRT might not significantly improve disease control but was favorably associated with low incidence of complications. The incidence of ocular toxicity was minimal and decreased vision was not observed. Late complications included xerophthalmia (1 patient), lacrimal stenosis (1 patient), and 1 patient developed an early cataract ~2 years after radiation treatment for an ethmoid sinus ENB. With a median follow-up time of 32.5 months, our data showed that severe late toxicities (grade 3 or 4) after IMRT were infrequent (11.5%), which included dysosmia (3.8%), hearing loss (3.8%), radiation brain injury (1.9%), and temporal lobe necrosis (1.9%). The incidence of ocular toxicity was minimal, and no patients experienced loss of vision. Of note, the reported median time for developing optic-nerve damage was 25 to 30 months (31).

The value of ENI has been suggested in a number of retrospective studies for locally advanced ENB. Early publications with small sample size questioned the necessity of ENI for patients with ENB (14, 32); however, modern series indicated otherwise. In a series of 67 cases ENB received 3-D conformal radiation therapy or IMRT with or without ENI after definitive surgery, 12% developed neck recurrence. However, none of the patients with neck recurrence received prophylactic neck radiation (15). Furthermore, in a more recently published study of 116 patients, ENI significantly reduced the risk of neck recurrence from 23 to 2% (28). Our findings seem to confirm the efficacy of ENI. Nearly all patients received ENI in our series, and only 2 patients (4.5%) of the 44 newly diagnosed patients developed neck recurrence. It is important to note that both patients had N1 disease at diagnosis. As the regional recurrence rate can be as high as 12–44% for locally advanced ENB and the outcome is usually dismal once recurrence occurs, we suggest a careful evaluation of the risk of nodal recurrence in patients with Kadish B and C patients.

Several pitfalls need to be discussed. First, as a retrospective study, the treatment regimens for patients included in this analysis were heterogeneous. Forty-four patients presented after initial diagnosis of ENB and 8 had either recurrent or secondary disease; 35 patients received surgery followed by IMRT, and 17 received IMRT without surgery as the treatment. In addition, chemotherapy was used in 28 patients at the discretion of the attending oncologists. The relatively limited number of patients due to the rarity of the disease, together with the mixed regimens used made it difficult to understand the role of individual treatment modality and their combinations. Ideally, well-designed prospective trials will be required to define the optimal treatment regimens; nevertheless, considering the rarity of the disease, it will be difficult to plan for a prospective clinical trial even with multi-institutional efforts. Currently, surgery followed by adjuvant radiotherapy or definitive radiation for unresectable/inoperable disease is the most utilized combination for ENB, with or without chemotherapy. Our results indicated that IMRT with ENI is efficacious and safe for both primary and recurrent/secondary ENB. Secondly, Hyams grade which reflect the biology ENB, is available for 12 patients only. Hyams grading was reported to correlate with treatment outcome. High grade ENB (i.e., Grade III or IV) were found to be related with more advanced local and regionally stages as well as worse survival outcome (33, 34). Unfortunately, we were not able to include Hyams grading in our uni- and multi-variate analyses due to the limited details of pathology reports. Last but not the least, the follow-up time of 32.5 months is relatively short for our cohort of patients, thus we could only report the 3-year survival and disease control outcome with confidence. Several researchers reported that recurrence occurs long (about 60 months) after the completion of ENB treatment (35). However, the pattern of recurrence had been reported as biphasic, with early recurrence at 17 months usually with poor prognosis, whereas patients with late recurrence enjoy a better prognosis (36).

Despite of the favorable OS and local/regional control rates, a number of issues remained puzzling. The role of chemotherapy has not been well-defined in the management of non-metastatic ENB. There is no standard regimen of chemotherapy for ENB, but cisplatin and etoposide seem to be the most acceptable combination used (37). The use of adjuvant chemotherapy may improve LPFS and RPFS but not OS (21). In a National Cancer Database (NCDB) analysis, chemotherapy improved efficacy of post-operative radiation therapy, especially in patients with Kadish C and D diseases (38). In our analyses, chemotherapy was used in nearly half of the patients. Although the regimen and timing of chemotherapy varied, no significant finding was observed in local, regional, or distant disease control. On univariate analysis using the log-rank test, patients receiving chemotherapy experienced worse LPFS (*p* = 0.032), potentially due to more advanced T-disease in patients who received chemotherapy (Table S6, *p* = 0.011). However, no significant association between chemotherapy and LPFS was observed on cox regression analysis (*p* = 0.293, Table S3). Clearly, efficacious chemotherapy regimens and combined treatment strategies need to be discovered then tested for ENB especially for patients with N+ diseases given the high probability of distant metastasis. In addition, the optimal dose of IMRT should also be further confirmed. Conventional radiation therapy was used in most published literatures on radiation for ENB. Whether higher radiation dose used in IMRT could further improve disease control thereby survival while maintaining a lower adverse effect profile is largely unknown. In our series, both uni- and multivariate analyses revealed that total dose of IMRT (above or below 66 Gy) was not significant for predicting OS, PFS, or local control rates. Nevertheless, with an LPFS of less than 90% in 3 years, further escalating of radiation dose should be investigated for a more optimal local control. A regional recurrence rate of 4.5% in our study for a group of patients largely with advanced or recurrent ENB indicated that ENI is effective in preventing neck recurrence.

## Conclusion

IMRT produced acceptable 3-year outcomes in terms of OS (89.7%), LPFS (89.7%), and RPFS (95.1%) rates without substantial late adverse effects. PFS remained at 69.5% due to, at least in part, a more suboptimal distant metastatic rate (85.4%). Further investigations for a more effective systemic regimen for distant disease control as well as a precision radiation technique for further improvement in local control will be needed.

## Data Availability Statement

The original contributions presented in the study are included in the article/Supplementary Material, further inquiries can be directed to the corresponding author/s.

## Ethics Statement

The studies involving human participants were reviewed and approved by the Institutional Review Board (IRB) of the Fudan University, Shanghai Cancer Center (FUSCC). The patients/participants provided their written informed consent to participate in this study.

## Author Contributions

CB: data acquisition, quality control of data and algorithms, data analysis and interpretation, statistical analysis, manuscript preparation, and manuscript review. WH and YD: data acquisition, data analysis and interpretation, manuscript preparation, and manuscript review. JH: data acquisition, data analysis and interpretation, statistical analysis, manuscript preparation, and manuscript review. JL and LK: study concepts, study design, quality control of data and algorithms, data analysis and interpretation, manuscript preparation, manuscript editing, and manuscript review. All authors contributed to the article and approved the submitted version.

## Conflict of Interest

The authors declare that the research was conducted in the absence of any commercial or financial relationships that could be construed as a potential conflict of interest.

## Abbreviations

IMRT: Intensity-modulated radiotherapy
ENB: Esthesioneuroblastoma
ENI: Elective nodal irradiation
OS: Overall survival
PFS: Progression-free survival
LPFS: Local progression-free survival
RPFS: Regional progression-free survival
DMFS: Distant metastasis-free survival
OARs: Organs at risk
IRB: Institutional review board
FUSCC: Fudan University, Shanghai Cancer Center
EP: Etoposide and cisplatin
BED: Biological equivalent dose
RTOG: Radiation Therapy Oncology Group
NCDB: National Cancer Database.
