# Supplementary material for: Intensity-Modulated Radiation Therapy for Esthesioneuroblastoma: 10-Year Experience of a Single Institute
Source: Front Oncol. 2020 Jul 17;10:1158. doi: 10.3389/fonc.2020.01158 (PMC7379860; doi:10.3389/fonc.2020.01158)
Supplement: Supplementary file 1 [file Data_Sheet_1.docx]

Supplementary Material

# Supplementary Figures and Tables

## Supplementary Figures

**
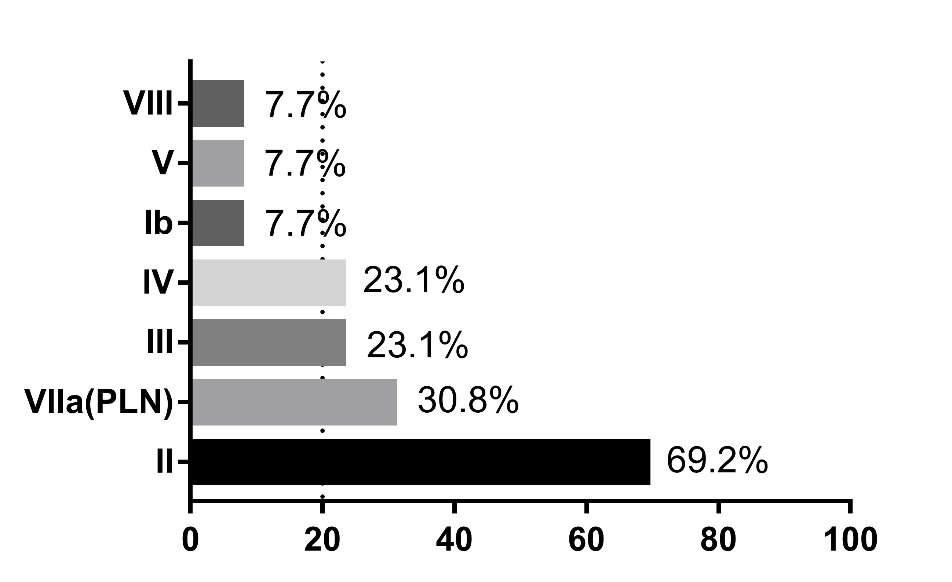
**

**Figure S1.** Distribution of involved neck lymph nodes in the 13 ENB patients with neck adenopathy.

## Supplementary Tables

**Table S1**. Univariate analyses of OS (Cox proportional hazards model)

| Variables | Univariate analyses | |  |
| --- | --- | --- | --- |
|  | HR (95% CI) | P value |  |
| Gender (female *vs.* male) | 1.384(0.154-12.470) | 0.772 |  |
| Age (continuous variable) | 1.025(0.956-1.098) | 0.489 |  |
| Recurrent ENB (no vs. yes) | 5.886(0.931-37.231) | *0.060* |  |
| **Salvage RT⁜ (no *vs.* yes)** | **3.345(0.554-20.200)** | **0.188** |  |
| Modified Kadish stage (A/B/C vs. D) | 0.698(0.078-6.287) | *0.749* |  |
| T-category (T1/2 *vs.* T3/4) | 0.366(0.041-3.295) | 0.370 |  |
| N-category (N- *vs.* N+)‡ | 0.698(0.078-6.287) | *0.749* |  |
| Surgery (No *vs.* Yes) | 0.761(0.126-4.607) | 0.766 |  |
| Chemotherapy* (No *vs.* Yes) | 0.281(0.031-2.530) | 0.258 |  |
| GTV dose (continuous variable) | 0.967(0.793-1.178) | 0.738 |  |
| Fractionation (continuous variable) | 0.001(0.000-884.925) | 0.328 |  |
| BED (continuous variable) | 0.967(0.824-1.136) | 0.684 |  |
| ENI (No *vs.* Yes) | 0.271(0.043-1.727) | 0.167 |  |

* Chemotherapy before IMRT and/or concurrent chemo-IMRT, and/or Chemotherapy after IMRT.

⁜ Salvage RT including 1 local recurrence after surgery and 7 re-irradiation patients

‡ Constant or Linearly Dependent covariates Modified Kadish stage (A/B/C vs. D) = N stage (N- vs. N+)

**Table S2**. Univariate analyses of PFS (Cox proportional hazards model)

| Variables | Univariate analyses | |  |
| --- | --- | --- | --- |
|  | HR (95% CI) | P value |  |
| Gender (female *vs.* male) | 1.196(0.332-4.308) | 0.784 |  |
| Age (continuous variable) | 1.018(0.979-1.059) | 0.372 |  |
| Recurrent ENB (no vs. yes) | 3.986(1.246-12.754) | *0.020* |  |
| **Salvage RT⁜ (no *vs.* yes)** | **3.009(1.007-8.991)** | ***0.049*** |  |
| Modified Kadish stage (A/B/C vs. D) | 3.295(1.154-9.408) | *0.026* |  |
| T-category (T1/2 *vs.* T3/4) | 0.764(0.256-2.286) | 0.631 |  |
| N-category (N- *vs.* N+)‡ | 3.295(1.154-9.408) | *0.026* |  |
| Surgery (No *vs.* Yes) | 0.608(0.211-1.754) | 0.357 |  |
| Chemotherapy* (No *vs.* Yes) | 1.340(0.463-3.882) | 0.589 |  |
| GTV dose (continuous variable) | 1.055(0.912-1.220) | 0.472 |  |
| Fractionation (continuous variable) | 0.017(0.000-115.925) | 0.364 |  |
| BED (continuous variable) | 1.038(0.924-1.165) | 0.533 |  |
| ENI (No *vs.* Yes) | 0.401(0.123-1.308) | 0.130 |  |

* Chemotherapy before IMRT and/or concurrent chemo-IMRT, and/or Chemotherapy after IMRT.

⁜ Salvage RT including 1 local recurrence after surgery and 7 re-irradiation patients

‡ Constant or Linearly Dependent covariates Modified Kadish stage (A/B/C vs. D) = N stage (N- vs. N+)

**Table S3**. Univariate analyses of LPFS (Cox proportional hazards model)

| Variables | Univariate analyses | |  |
| --- | --- | --- | --- |
|  | HR (95% CI) | P value |  |
| Gender (female *vs.* male) | 33.247(0.003-396682.698) | 0.464 |  |
| Age (continuous variable) | 1.034(0.956-1.119) | 0.398 |  |
| Recurrent ENB (no vs. yes) | 3.811(0.395-36.783) | 0.247 |  |
| **Salvage RT⁜ (no *vs.* yes)** | **5.840(0.821-41.522)** | ***0.078*** |  |
| Modified Kadish stage (A/B/C vs. D) | 1.012(0.105-9.767) | 0.992 |  |
| T-category (T1/2 *vs.* T3/4) | 4.285(0.444-41.392) | 0.209 |  |
| N-category (N- *vs.* N+)‡ | 1.012(0.105-9.767) | 0.992 |  |
| Surgery (No *vs.* Yes) | 1.320(0.137-12.697) | 0.810 |  |
| Chemotherapy* (No *vs.* Yes) | 76.203(0.024-246565.782) | 0.293 |  |
| GTV dose (continuous variable) | 1.011(0.787-1.298) | 0.932 |  |
| Fractionation (continuous variable) | 0.005(0.000-17336.373) | 0.494 |  |
| BED (continuous variable) | 1.002(0.820-1.224) | 0.985 |  |
| ENI (No *vs.* Yes) | 0.136(0.019-0.964) | ***0.046*** |  |

* Chemotherapy before IMRT and/or concurrent chemo-IMRT, and/or Chemotherapy after IMRT.

⁜ Salvage RT including 1 local recurrence after surgery and 7 re-irradiation patients

‡ Constant or Linearly Dependent covariates Modified Kadish stage (A/B/C vs. D) = N stage (N- vs. N+)

**Table S4**. Univariate analyses of RPFS (Cox proportional hazards model)

| Variables | Univariate analyses | |  |
| --- | --- | --- | --- |
|  | HR (95% CI) | P value |  |
| Gender (female *vs.* male) | 32.702(0.000-24303617.22) | 0.613 |  |
| Age (continuous variable) | 0.963(0.871-1.065) | 0.461 |  |
| Recurrent ENB (no vs. yes) | 0.043(0.000-52743305.50) | 0.768 |  |
| **Salvage RT⁜ (no *vs.* yes)** | **0.039(0.000-715772.328)** | **0.703** |  |
| Modified Kadish stage (A/B/C vs. D) | 352.358(0.000-2.282E9) | 0.464 |  |
| T-category (T1/2 *vs.* T3/4) | 1.415(0.088-22.643) | 0.806 |  |
| N-category (N- *vs.* N+)‡ | 352.358(0.000-2.282E9) | 0.464 |  |
| Surgery (No *vs.* Yes) | 0.460(0.029-7.355) | 0.583 |  |
| Chemotherapy* (No *vs.* Yes) | 0.954(0.059-15.318) | 0.974 |  |
| GTV dose (continuous variable) | 1.455(0.912-2.322) | 0.115 |  |
| Fractionation (continuous variable) | 0.001(0.000-5186776.010) | 0.522 |  |
| BED (continuous variable) | 1.302(0.904-1.874) | 0.156 |  |
| ENI (No *vs.* Yes) | 24.353(0.000-3.467E9) | 0.739 |  |

* Chemotherapy before IMRT and/or concurrent chemo-IMRT, and/or Chemotherapy after IMRT.

⁜ Salvage RT including 1 local recurrence after surgery and 7 re-irradiation patients

‡ Constant or Linearly Dependent covariates Modified Kadish stage (A/B/C vs. D) = N stage (N- vs. N+)

**Table S5**. Univariate analyses of DMFS (Cox proportional hazards model)

| Variables | Univariate analyses | |  |
| --- | --- | --- | --- |
|  | HR (95% CI) | P value |  |
| Gender (female *vs.* male) | 0.325(0.066-1.613) | 0.169 |  |
| Age (continuous variable) | 1.013(0.955-1.073) | 0.673 |  |
| Recurrent ENB (no vs. yes) | 5.373(0.981-29.426) | *0.053* |  |
| **Salvage RT⁜ (no *vs.* yes)** | **2.692(0.492-14.716)** | **0.253** |  |
| Modified Kadish stage (A/B/C vs. D) | 3.062(0.618-15.185) | 0.171 |  |
| T-category (T1/2 *vs.* T3/4) | 0.299(0.035-2.566) | 0.271 |  |
| N-category (N- *vs.* N+)‡ | 3.062(0.618-15.185) | 0.171 |  |
| Surgery (No *vs.* Yes) | 0.938(0.172-5.123) | 0.941 |  |
| Chemotherapy* (No *vs.* Yes) | 0.491(0.090-2.692) | 0.413 |  |
| GTV dose (continuous variable) | 0.970(0.802-1.174) | 0.755 |  |
| Fractionation (continuous variable) | 0.001(0.000-568.571) | 0.300 |  |
| BED (continuous variable) | 0.970(0.831-1.131) | 0.695 |  |
| ENI (No *vs.* Yes) | 0.683(0.080-5.860) | 0.728 |  |

* Chemotherapy before IMRT and/or concurrent chemo-IMRT, and/or Chemotherapy after IMRT.

⁜ Salvage RT including 1 local recurrence after surgery and 7 re-irradiation patients

‡ Constant or Linearly Dependent covariates Modified Kadish stage (A/B/C vs. D) = N stage (N- vs. N+)

**Table S6** Baseline characteristics between patients treated with or without chemotherapy 52 patients with Esthesioneuroblastoma

|  | With Chemotherapy  (28 patients) | | Without Chemotherapy  (24 patients) | | p-value  (2-sided) |
| --- | --- | --- | --- | --- | --- |
|  | n | % | n | % |  |
| Gender |  |  |  |  | 0.190 |
| Male | 24 | 85.7 | 17 | 70.8 |  |
| Female | 4 | 14.3 | 7 | 29.2 |  |
| Age (years) |  |  |  |  | 0.331 |
| Median(range) | 44 (18-74) | | 49(28-71) | |  |
| Modified Kadish stage |  |  |  |  | 0.106 |
| A | 1 | 3.6 | 3 | 12.5 |  |
| B | 6 | 21.4 | 11 | 45.8 |  |
| C | 11 | 39.3 | 5 | 20.8 |  |
| D* | 10 | 35.7 | 5 | 20.8 |  |
| T-classification |  |  |  |  | ***0.011*** |
| 1 | 3 | 10.7 | 10 | 41.7 |  |
| 2 | 10 | 35.7 | 8 | 33.3 |  |
| 3 | 5 | 17.9 | 5 | 20.8 |  |
| 4 | 10 | 35.7 | 1 | 4.2 |  |
| N-classification |  |  |  |  | 0.238 |
| 0 | 18 | 64.3 | 19 | 79.2 |  |
| 1 | 10 | 35.7 | 5 | 20.8 |  |
| Surgery |  |  |  |  | 0.053 |
| R0/R1 | 4 | 14.3 | 8 | 33.3 |  |
| R2 | 11 | 39.3 | 12 | 50 |  |
| Biopsy | 13 | 46.4 | 4 | 16.7 |  |
| IMRT |  |  |  |  | 1.000 |
| primary IMRT | 24 | 85.7 | 20 | 83.3 |  |
| Salvage IMRT | 4 | 14.3 | 4 | 16.7 |  |
| Total dose of IMRT, Gy |  |  |  |  | 0.060 |
| Median (range) | 66 (52.5-75) | | 66 (56-70) | |  |
| Fractionation of IMRT, Gy |  |  |  |  | 0.657 |
| Median (range) | 2 (2-2.2) | | 2 (1.8-2.2) | |  |
| ENI |  |  |  |  | 1.000 |
| No | 3 | 10.7 | 3 | 12.5 |  |
| Yes | 25 | 89.3 | 21 | 87.5 |  |
| Severe late toxicities |  |  |  |  | 0.815 |
| No | 24 | 85.7 | 22 | 91.7 |  |
| Yes | 4 | 14.3 | 2 | 8.3 |  |

* All had regional lymphadenopathy without distant metastasis. Abbreviation: IMRT – intensity-modulated radiation therapy.
